# Improved Diet Quality and Nutrient Adequacy in Children and Adolescents with Abdominal Obesity after a Lifestyle Intervention

**DOI:** 10.3390/nu10101500

**Published:** 2018-10-13

**Authors:** Ana Ojeda-Rodríguez, Itziar Zazpe, Lydia Morell-Azanza, María J. Chueca, Maria Cristina Azcona-sanjulian, Amelia Marti

**Affiliations:** 1 Department of Nutrition, Food Sciences and Physiology, University of Navarra. C/ Irunlarrea, 1. 31008 Pamplona, Spain; aojeda.5@alumni.unav.es (A.O.-R.); lmorell.1@alumni.unav.es (L.M.-A.); 2 IdiSNA, Instituto de Investigación Sanitaria de Navarra. C/Irunlarrea, 3. 31008 Pamplona, Spain; mchuecag@cfnavarra.es (M.J.C.); cazcona@unav.es (M.C.A.-S.); 3 Department of Preventive Medicine and Public Health, School of Medicine-Clínica Universidad de Navarra, University of Navarra. C/ Irunlarrea, 1. 31008 Pamplona, Spain; 4 Biomedical Research Centre Network on Obesity and Nutrition (CIBERobn), Physiopathology of Obesity and Nutrition, Institute of Health Carlos III. Av. Monforte de Lemos, 3-5. 28029 Madrid, Spain; 5 Paediatric Endocrinology Unit, Complejo Hospitalario de Navarra. C/Irunlarrea, 3. 31008 Pamplona, Spain; 6 Paediatric Endocrinology Unit, Department of Paediatrics. Clínica Universidad de Navarra. Av. Pío XII, 36. 31008 Pamplona, Spain

**Keywords:** dietary intervention, childhood obesity, Mediterranean diet, nutritional requirements

## Abstract

High rates of childhood obesity require integral treatment with lifestyle modifications that achieve weight loss. We evaluated a lifestyle intervention on nutrient adequacy and diet quality in children and adolescents with abdominal obesity. A randomized controlled trial was performed on 107 participants, assigned either to a usual care group or to an intensive care group that followed a moderate hypocaloric Mediterranean diet and received nutritional education. Intake adequacy was evaluated using Dietary Reference Intakes and diet quality through the Diet Quality Index for Adolescents (DQI-A), the Healthy Lifestyle Diet-Index (HLD-I) and the Mediterranean Diet Quality Index (KIDMED). Both groups achieved a significant reduction in BMI standard deviation score (BMI-SDS), glucose and total cholesterol levels. Intake of Calcium, Iodine and vitamin D were higher in the intensive care group, with enhanced compliance with recommendations. Higher dietary scores were associated with lower micronutrient inadequacy. DQI-A and HLD-I were significantly higher in the intensive care group vs. usual care group after the treatment. In conclusion, we observed that an intensive lifestyle intervention was able to reduce BMI-SDS in children with abdominal obesity. Furthermore, participants significantly improved dietary indices getting closer to the nutritional recommendations. Therefore, these diet quality indices could be a valid indicator to evaluate micronutrient adequacy.

## 1. Introduction

Obesity is a multifactorial disease and its treatment requires a multidisciplinary approach. Although weight gain could have a genetic component, lifestyle factors are the most important modifiable risk factors [1,2]. In recent years, there has been an increase in portion size and consumption of high-energy foods and a decrease in fruit and vegetable consumption in paediatric populations, in parallel with a reduction in physical activity levels and a rise in sedentary behaviour [3]. Intervention studies able to change lifestyle habits and improve diet quality are considered key in the treatment of paediatric obesity [4,5,6]. Several lifestyle interventions were able to lower anthropometric and cardiometabolic risk factors [7,8,9,10], but few interventions have considered changes in diet quality and nutrient adequacy as a final outcome [11,12]. On the other hand, dietary indices could be a useful tool to identify subjects at high nutritional risk, being those with a poor diet and lifestyle pattern [13,14,15,16]. For this reason, the aim of our study was to evaluate, in children with abdominal obesity, the effect of a lifestyle intervention on nutrient adequacy and diet quality, as assessed by Diet Quality Index for Adolescents (DQI-A), Healthy Lifestyle Diet-Index (HDL-I) and Mediterranean Diet Quality Index (KIDMED).

## 2. Materials and Methods

### 2.1. Participants

The IGENOI study is a two-year family-based lifestyle intervention study involving children and adolescents with abdominal obesity. It is a randomized controlled clinical trial (NCT03147261) carried out in Pamplona by members of the GENOI group (Navarro Study Group of Childhood Obesity). Participants were recruited at the Paediatric Endocrinology Unit of the Clinic University of Navarra, the Paediatric Department of the University Hospital Complex of Navarra and Health Centres in Pamplona. The study population comprised children aged seven to 16 years, with a waist circumference above the 90th percentile according to national data [17]. The exclusion criteria included prevalent pre-diabetes, food intolerance, eating disorders or psychiatric disease, pharmacological treatment, regular alcohol consumption or special diet treatment. The study followed the ethical standards recognized in the 2013 Declaration of Helsinki and was approved and supervised by the Human Research Ethics Committee of the University of Navarra (044/2014). Written informed consent was obtained from eligible children and their parents or legal guardians.

A total of 126 children were initially recruited. Among them, four did not meet the inclusion criteria and eight dropped out after random assignment due to discouragement, time incompatibility, social problems or a change in phone number or e-mail address. Thus, although 114 participants concluded the eight-week phase (dropout rate: 6.5%), 109 participants completed the dietary intake data at baseline and after the intervention. Two participants were identified as outliers given their high baseline energy intake values and were removed from the analysis [10,18]; thus, the final subsample was 107 children.

### 2.2. Experimental Design

This multidisciplinary intervention consisted of a two-year programme that comprises an eight-week phase with a total follow-up period of 22 months. A multidisciplinary team, including registered dieticians, paediatricians, physical activity experts and nurses, carried out the intervention. In this study, we present data from the treatment period corresponding to the first eight weeks, since the study is still ongoing.

Subjects were randomly assigned to the usual or intensive care group at a ratio of 1:3. They were accompanied by their parents or legal guardians. The usual group received standard paediatric recommendations [19] on healthy diet, while the intensive care group was advised to follow a moderately hypocaloric Mediterranean diet [9,20]. Both groups were encouraged to accumulate an extra time of 200 min of physical activity per week at 60–75% of their maximum heart rate.

During the eight-week period, usual care subjects received a 30-min individual session with the dietician and five monitoring visits to assess anthropometric parameters. Participants assigned to the intensive care group completed six 30-min individual sessions with the research team.

In addition, intensive care subjects had one group session. Dieticians provided information on healthy lifestyle and how to manage obesity-related problems to parents and legal guardians. On the other hand, children were taught several topics such as controlling healthy lifestyle behaviour, food preparation, portion control, eating behaviour, food composition and the importance of being physically active (by increasing physical activity and reducing sedentary time) during leisure time.

### 2.3. Dietary Intervention

On the first visit, intensive care participants were prescribed to follow a diet based on a fixed full-day meal plan. Their energy expenditure was calculated using the Schofield equation adapted to age and sex [21]. The percentage of calorie restriction varied from 10% to 40% of total energy intake depending on the degree of obesity presented [20], which did not interfere with the children’s body growth. In all cases, the caloric ranges of diets were between 1300 and 2200 kcal.

The diet was distributed into five main meals: breakfast providing 20%, morning snack 5–10%, lunch 30–35%, afternoon snack 10–15% and dinner 20–25% of total energy. The distribution of carbohydrate, fat and proteins was 55%, 30% and 15% of total energy, respectively [22]. Intensive care participants followed a Mediterranean-style diet based on high consumption of fruit, vegetables, whole grains, legumes, nuts, seeds and olive oil, minimally processed foods; moderate consumption of dairy products, fish and poultry; and low consumption of red meat [23].

### 2.4. Anthropometric, Clinical and Biochemical Measurements

Trained personnel carried out all anthropometric measurements using calibrated equipment, at both the beginning and end of the eight-week period. Participants were barefoot and wearing light clothes.

Height was obtained with a Harpenden’s stadiometer of 1 mm precision (Seca 220, Vogel & Halke, Hamburg, Germany) and body weight using a digital scale (BC-418, TANITA, Tokyo, Japan). Body Mass Index (BMI) was calculated from the ratio of weight to height squared (kg/m^2^) and converted after into standard deviation scores (SDS) for sex and age derived from Spanish reference data according to specific cutoff points for BMI [17]. Finally, pubertal development was evaluated according to Tanner stages [24].

Venous blood samples were obtained by specialized trained nurses at the hospital after an overnight fast. Glucose, insulin and total cholesterol were determined by standard autoanalyzer techniques.

Blood pressure was measured using an electronic sphygmomanometer (OMRON M6, Hoofddorp, The Netherlands) on the right arm after the children had rested quietly for 15 min.

### 2.5. Physical Activity

Physical activity during leisure time and sedentary behaviour was objectively measured over four consecutive full days, two weekdays and two weekend days, by triaxial accelerometry (Actigraph wGT3X-BT, Actigraph LLC, Penascola, FL, USA). The accelerometer collected data in 60-s intervals [25], which were analysed using ActiLife 6.0 software (Actigraph LLC, Penascola, FL, USA), and summarized as counts per min (CPM) using validated cutoff points to define time spent sedentary (<100 CPM) and moderate-to-vigorous physical activity (MVPA) (>2296 CPM) [26].

### 2.6. Dietary Intake Assessment

Trained dieticians collected dietary intake data at baseline and after an eight-week period using a baseline semi-quantitative 136-item Food-Frequency Questionnaire (FFQ) [20], which was previously validated in Spain and recently re-evaluated [27,28]. We also examined diet quality through three dietary indices: DQI-A, HDL-I and KIDMED. The dietary indices provide different information about the diversity of the diet (DQI-A), lifestyle habits such as physical activity (HDL-I) and adherence to the Mediterranean diet (KIDMED). In all cases, higher values of each index indicated a greater diet quality.

The DQI-A has been previously validated and is adapted for use in adolescents [13]. The overall DQI-A score is composed of the sum of three categories (quality, diversity and equilibrium), presented in percentages. Its total value ranges from ‒3% to 100%. To evaluate daily intake, the original DQI-A considered nine recommended groups: (1) water, (2) bread and cereals, (3) potatoes and grains, (4) vegetables, (5) fruits, (6) milk products, (7) cheese, (8) meat, fish and substitutes, (9) fats and oils. However, we modified the original DQI-A and divided it into eight recommended groups because data on water were not collected. DQI-A has been previously described in more detail [29].

The HDL-Index is composed of 10 items, eight of which refer to the frequency of consumption of fruit, vegetables, fish and seafood, sweets, regular soft drinks, grain, dairy products, meat and meat products. The other components indicate the level of physical activity through measuring the time spent on moderate to vigorous physical activity versus looking at screens [14]. The HDL-Index employed in this study is a modified version of the original index due to a lack of information on screen hours. Thus, the range of this index was between 0 and 36.

Finally, KIDMED index score evaluated the adequacy of Mediterranean dietary pattern in children and adolescents and ranged from 0 to 12 [30].

### 2.7. Statistical Analysis

The sample size calculation indicated that 13 and 39 subjects were needed for usual and intervention care group, respectively. This estimation was based on the following assumptions: an error of 5%, a power of 90%, a 1:3 ratio, and a mean difference of 0.50 (SD 0.47) units in BMI-SDS after the nutritional intervention, following similar criteria previously applied by members of our team project [31,32]. Student’s unpaired or paired *t*-test were used for comparison between groups or within group changes, respectively. The differences in the changes of variables between Usual care group and Intensive care group were assessed using analysis of covariance (ANCOVA) adjustment for potential confounders such as BMI-SDS, total energy intake or corresponding variables at baseline (data not shown), however similar results were obtained. The inter-quartile rank was used for outlier detection; values outside the 1.5 limits for energy intake were identified as outliers and were removed [33].

To evaluate prevalence of micronutrient adequacy, we used the Dietary Reference Intakes (DRIs) and the methods endorsed by the Institute of Medicine [34]. Thus, we used Estimated Average Requirement (EAR) or Adequate Intake (AI) if the EAR values were not available, and Upper Levels (UP) to examine the adequacy of 19 micronutrients: Ca, Fe, I, Mg, Zn, Na, K, P, Se, and vitamins B1, B2, B3, B6, B12, C, A, D, E and folic acid. Regarding macronutrients, we used the Acceptable Macronutrients Distribution Ranges (AMDR) [35]. The prevalence of subjects with inadequate intake between the usual and intensive care groups was compared using the McNemar test.

To test associations between inadequacy and indexes we performed a score consisting of the addition of inadequacy (%) in those subjects with nutrients < EAR. Test of linear trend across successive tertiles of each index were performed assigning the median value to each tertile and treating the variables as continuous.

STATA 12.0 for Windows (version 12.0, College Station, TX: StataCorp LP, USA) was used as statistical software. The statistical significance level was *p* < 0.05.

## 3. Results

Clinical parameters, diet quality and nutrient adequacy data were collected from 107 children with abdominal obesity (11.3 years old, 63% females). As expected, subjects from usual care (*n* = 26) and intensive care (*n* = 81) groups had similar baseline clinical measurements, except for glucose levels (Table 1). Thus, the usual care group had significantly higher glucose levels compared with the intensive care group (*p* = 0.034). Food and nutrient intakes and diet quality indices were also comparable.

Changes in clinical parameters and food intake after the lifestyle intervention are described in Table 1. Participants of both groups showed a significant decrease in body weight, BMI-SDS, glucose and total cholesterol levels. After the intervention, they significantly reduced consumption of sausages, refined grains and sweets, while increased whole grain consumption. In addition, the intensive care group significantly reduced insulin levels, blood pressure and meat intake, and they increased consumption of fruits, vegetables, dairy products and fish in comparison with usual care group. In the intensive care group, moderate-to-vigorous physical activity time significantly increased after the intervention.

As shown in Table 1, the change in glucose levels between group was statistically significant (*p* = 0.033), but did not remain statistically significant in ANCOVA model when it was adjusted for baseline BMI-SDS and glucose levels (*p* = 0.367, data not shown).

Table 2 shows the changes in daily macro- and micronutrient intakes after the intervention. As a consequence of the reduction in total energy intake, both groups significantly reduced the intake of all macronutrients and cholesterol, Fe, Zn, Na and vitamin E intake. In addition, intensive care subjects had greater intake of fibre, I, vitamin B12, vitamin C, and vitamin D intake after the intervention.

The percentage of energy from macronutrients and the changes in the proportion of individuals within the AMDR at baseline and after the intervention are shown in Table 3. A similar reduction in total energy intake was found in both groups (usual care subjects −766 kcal/d and intensive care subjects −731 kcal/d). However, the usual care group significantly increased protein (+3% of total energy) and decreased fat intake (−3% of total energy) after the intervention, resulting in a 27% increase in fat AMDR of participants. Notably, intensive care subjects increased carbohydrates and protein intake, and decreased fat content, in this way, the percentage of participants within AMDR for fat was improved (38%).

**Figure 1:**
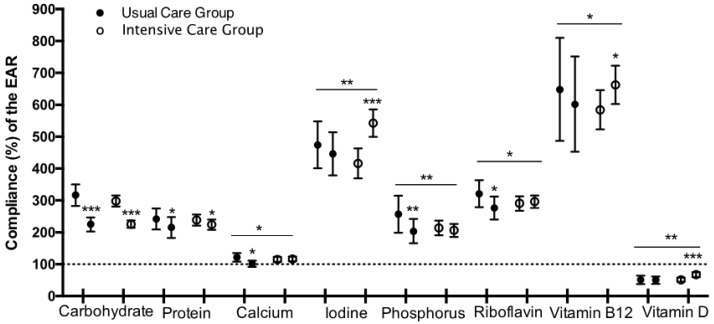
Change in the compliance (%) of the dietary recommendations (EAR) after lifestyle intervention in children with abdominal obesity. * *p*-value < 0.05, ** *p*-value < 0.01 and *** *p*-value < 0.001.

Moreover, we evaluated compliance with macro- and micronutrients EAR (%). Only those micronutrients with significant changes between groups after the intervention are displayed in Figure 1. Despite all participants not being within the carbohydrate AMDR, they reached values closer to the carbohydrate and protein recommendations. Although all micronutrients were above EAR (except vitamin D), usual care subjects, unlike the intensive care group, diminished compliance for Ca, P and vitamin B2 intake. On the contrary, intensive care subjects enhanced their compliance with I and vitamin B12 intake.

It is noteworthy that vitamin D was under EAR in both groups, and intensive care subjects significantly improved their compliance (51% to 61%, *p* < 0.001). Dietary inadequacy for 19 micronutrients is shown in Table 4. The prevalence of subjects above Na UL decreased after the intervention (−38% for the usual care group and −47% for the intensive care group). Regarding vitamins, inadequate intakes were observed for vitamin D in most participants (88% of usual care subjects and 92% of intensive care subjects) at baseline. It is important to highlight that this percentage was significantly reduced (−21%) in intensive care subjects after the intervention. We found differences between groups in the proportion of individuals with inadequate vitamin A intake, with a favourable change in the intensive care group. The prevalence of participants with inadequate vitamin E intake significantly increased (35% in the usual care group and 32.1% of the intensive care group). As mentioned above, changes in the quality of diet were evaluated with three dietary indices: DQI-A with the categories: dietary quality, dietary diversity and dietary equilibrium, HDL-I and KIDMED (Table 5). Both groups significantly enhanced total DQI-A and two out of three categories: dietary quality and dietary equilibrium, although the increase in total DQI-A was higher in the intensive care group. The intensive care group significantly improved HLD-I score in 4.1 points vs. 1.4 points in the usual care group. In the same way, the KIDMED was two points and three points higher in usual care and intensive care subjects, respectively.

**Figure 2:**
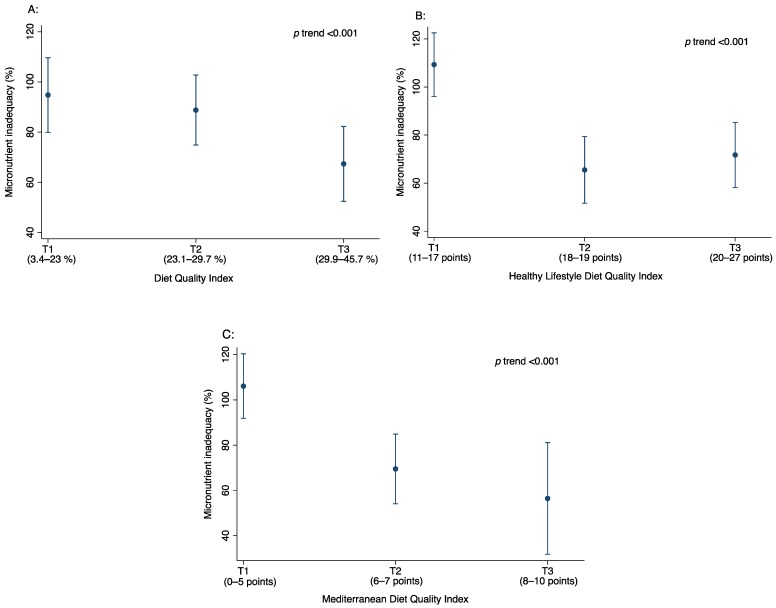
Micronutrient inadequacy (%) across the tertiles (T) of Diet Quality Index (**A**), Healthy Lifestyle Diet Index (**B**) and Mediterranean Diet Quality (**C**) at baseline in children with abdominal obesity.

Interestingly, the intensive care group presented a significant change in total DQI and HLD-I compared to the usual care group. Finally, when we analysed the association between tertiles of each diet quality index and inadequacy intake in the total population, we found that the sum of inadequacy percentage was lower in the higher tertile of total DQI-A, HLD-I and KIDMED with a significant trend (*p* < 0.001) (Figure 2).

## 4. Discussion

In the present work, we evaluated the effects of a lifestyle intervention on nutrient adequacy and diet quality in a population of children and adolescents with abdominal obesity. Participants were able to reduce their BMI-SDS (−0.5 units) and improve their diet quality scores, getting closer to the nutritional recommendations. Importantly, changes in BMI-SDS (≥−0.5) are associated with an improvement in cardiometabolic risk, as seen in our intervention [36,37].

The intensive care group did increase dairy products and fish intakes together with a reduced intake of meat and sausages, compared with the usual care group. Consequently, intakes of I, Ca and vitamin D were also higher in intensive care subjects, with enhanced compliance with recommendations and nutrient adequacy. Despite Iodine playing a key role in psychomotor growth during puberty, its deficiency is common in children [38]. To our knowledge, this is the first intervention study in which there is an increase in I intake from higher daily intakes of dairy products and fish.

Adequate intake of Ca and vitamin D are needed as the peak bone mass is achieved right after puberty [39,40]. In Spain, vitamin D deficiency is a serious health problem with a suboptimal intake in a high percentage of children [41,42,43]. We observed that vitamin D is the only micronutrient for which 100% of the DRI is not met, with a high number of subjects under EAR. After the intervention, we demonstrated that subjects were able to increase vitamin D intake enhancing the compliance of DRI through a dietary plan and nutritional education.

The usual intake of Na exceeded the ULs in a high number of participants in our study, similar to the findings reported in other Spanish children populations [43]. Salt-added foods (e.g., pre-cooked foods) are the main source of Na [44], which is associated with a risk of hypertension in children and adolescents [45,46]. We showed a decrease in Na intake, with a lower number of subjects above ULs in both groups. In contrast, Couch et al. [47] reported that a three-month behavioural intervention in overweight children did not reduce Na intake.

Notably, all subjects were within AMDR for protein intake (10–30% of total energy) before and after the intervention, although they exceeded the protein EAR at baseline. This observation is in line with the results of a national sample of Spanish children, which presented an excess of protein EAR, although they met the AMDR for protein intake [42]. Unfortunately, 45% and 38% of participants had carbohydrate and fat intake outside AMDR, respectively, similar to the data of a national sample of Spanish normal-weight children and adolescents [42,48]. Similar results were observed in overweight Greek children who consumed less energy from carbohydrates and more from fat according to AMDR [49].

After the intervention, all subjects (usual and intensive care groups) had improved macronutrient distribution and got closer to the nutritional recommendations. Similarly, Nemet et al. showed an improvement in carbohydrate and fat intake after a lifestyle intervention in obese children and adolescents [50], as found in our intervention.

Lifestyle and dietary indices are appropriate tools that provide overall diet quality [15]. Thus, they showed associations with healthy lifestyle patterns [13,14,15,51,52] and effective obese treatment [53] in children. At baseline, we observed that higher scores of diet quality indices were associated with lower nutrient inadequacy intake. This suggests that these indices could be used as valid indicators to evaluate micronutrient adequacy.

Thus, after the intensive lifestyle intervention, both usual and intensive care groups presented beneficial changes in the three indices: DQI-A, HLD-I and KIDMED. As expected, we observed a greater improvement in the intensive care group. This indicates the accomplishment of a healthy dietary pattern and physical activity recommendations after the intervention, which indicates good adherence to the treatment.

Vyncke et al. [13] observed a positive association between DQI-A and nutrient-dense food, and a negative association with energy-dense and low-nutrient foods in European adolescents.

In line with our research, Manios et al. [14] found that higher HLD-I scores were associated with lower micronutrient inadequacy intakes according to EAR. Furthermore, adapted versions of HLD-I showed inverse associations with weight status [54] and insulin resistance in normal-weight [14] and overweight children [55].

In paediatric populations, adherence to the Mediterranean diet has been associated with the prevention or treatment of childhood obesity [56]. Recently, it has been indicated that the KIDMED index has a direct association with lifestyle factors (physical activity and diet adequacy) [57]. In our intervention, the intensive care group showed an increase (42%) in Mediterranean Diet adherence, with an average final KIDMED score corresponding to optimal diet quality. In line with our results, Ranucci et al. [58] observed improvement in KIDMED scores (30%) in a multidisciplinary intervention in overweight/obese children and adolescents. In addition, Serra-Majem et al. [59] demonstrated that a higher adequacy to the Mediterranean Diet was associated with decreased percentages of inadequate micronutrients in subjects aged 6‒24 years old.

The main strengths of our study are the following: (a) the longitudinal design provides the possibility of making paired comparisons with baseline data used as control, (b) the involvement of registered nutritionists to collect dietary data, and (c) the good response of participants, which resulted in substantial weight loss and measurable changes in lifestyle factors.

We acknowledge that the present study has several limitations. First, there were different ages and pubertal stages among the participants. To minimize this effect, the DRIs used to analyse diet quality and nutrient adequacy were adjusted for sex and age. Second, the original DQI-A and HDL-I were modified slightly because of a lack of information on water consumption and hours of television viewing for all subjects. However, weight loss was substantial and several changes in lifestyle parameters were statistically significant. However, larger studies with long-term follow-up are necessary to corroborate our findings.

In conclusion, we showed that an intensive lifestyle intervention in children and adolescents with abdominal obesity achieved a reduction in BMI-SDS and improved adherence to nutritional recommendations assessed by diet quality indices. In addition, higher diet quality scores were found to be associated with higher micronutrient adequacy.

## Figures and Tables

**Table 1:** Changes in clinical and dietary parameters after lifestyle intervention in children with abdominal obesity.

	Baseline				
			Changes within group after eight weeks		
	Usual Care Group (*n* = 26)	Intensive Care Group (*n* = 81)	Usual Care Group (*n* = 26)	Intensive Care Group (*n* = 81)	Changes between Groups (Intensive vs. Usual Care)
	Mean (SD) or Percentage		Mean (95% CI)		Mean (95% CI)
**Age (years)**	10.7 (2.4)	11.49 (2.5)			
**Sex (F)**	18 (69)	49 (61)			
**Tanner (1/2/3/4/5)**	1(38) 2(8) 3(15) 4(4) 5(23)	1(33) 2(19) 3(14) 4(6) 5(25)			
**Weight (kg)**	62.7 (17.9)	67.27 (19.7)	−2.2 (−3.1, −1.3) ***	−2.6 (−3.1, −2.2) ***	−0.4 (−1.4, 0.5)
**Height (cm)**	147.9 (12.9)	151.75 (13.3)	1.2 (0.9, 1.5) ***	0.9 (0.8, 1.0) ***	−0.3 (−0.6, −0.00) *
**BMI**	28.1 (4.5)	28.52 (4.6)	−1.4 (−1.8, −0.9) ***	−1.5 (−1.7, −1.3) ***	−0.1 (−0.6, 0.3)
**BMI-SDS**	2.9 (1.2)	2.86 (1.0)	−0.5 (−0.8, −0.2 ) **	−0.5 (−0.6, −0.4) ***	−0.0 (−0.4,0.2)
**Glucose (mg/dL)**	91.2 (6.05)	88.0 (6.3) *	−5.8 (−8.9, −2.7) ***	−2.0 (−3.8, −0.3) *	3.8 (0.3, 7.2) *
**Insulin (µU/mL)**	19.9 (20.5)	16.3 (8.5)	−3.5 (−8.3, 1.2)	−2.1 (−3.7, −0.6) **	1.4 (−2.4, 5.1)
**Total Cholesterol (mg/dL)**	156.8 (22.6)	164.8 (27.3)	−11.4 (−18.4, −4.4) **	−11.9 (−17.2, −6.6) ***	−0.6 (−10.3, 9.2)
**Systolic BP (mmHg)**	113.3 (11.5)	119.0 (12.0) *	0.76 (−4.6, 6.2)	−6.6 (−9.0, −4.3) ***	−7.4 (−12.5, −2.3) **
**Diastolic BP (mmHg)**	71.5 (8.1)	72.8 (8.2)	1.68 (−4.2, 7.5)	−3.5 (−5.4, −1.6) ***	−5.2 (−9.8, −0.6) *
**Fruits (g/day)**	254.0 (135.5)	253.4 (177.3)	9.4 (−78.9, 97.8)	70.4 (20.6, 120.2) **	61.0 (−39.1, 161.0)
**Vegetables (g/day)**	262.0 (156.1)	313.1 (162.9)	60.0 (−31.7, 151.7)	68.1 (28.3, 107.8) **	8.1 (−77.7, 93.9,)
**Legumes (g/day)**	17.6 (10.2)	20.2 (11.1)	−1.3 (−5.6, 2.9)	0.7 (−2.2, 3.6)	2.0 (−3.6, 7.6,)
**Dairy Products (g/day)**	497.4 (198.3)	457.1 (192.7)	−83.3 (−181.9, 15.3)	93.6 (43.2, 144.0) ***	176.9 (73.3, 280.5) **
**Meat (g/day)**	180.0 (44.9)	205.8 (60.1) *	−23.2 (−47.3, 1.0)	−53.6 (−68.4, −38.9) ***	−30.5 (−59.6, −1.3,) *
**Sausages (g/day)**	9.7 (11.0)	21.1 (21.6) *	−5.5 (−10.5, −0.6) *	−19.0 (−23.6, −14.4) ***	−13.5 (−22.1, −4.9,) **
**Fish (g/day)**	70.2 (42.4)	71.2 (36.3)	12.7 (−2.4, 27.9)	31.7 (22.5, 40.9) ***	19.0 (0.8, 37.1) *
**Whole Grains (g/day)**	3.7 (10.5)	21.3 (54.3)	48.6 (17.1, 80.0) **	28.0 (11.4, 44.6) **	−20.6 (−54.5, 13.3,)
**Refined Grains (g/day)**	181.31 (76.3)	163.5 (92.8)	−97.2 (−126.9, −67.5) ***	−74.4 (−95.9, −52.9) ***	22.8 (8.4, −64.0)
**Eggs (g/day)**	23.4 (10.9)	24.3 (9.8)	−1.1 (−5.3, 3.1)	−0.7 (−2.8, 1.3)	0.4 (−3.9, 4.7)
**Nuts (g/day)**	0.1 (0.1)	0.1 (0.1)	0.4 (−1.2, 2.0)	−0.9 (−1.7, 0.0)	−1.3 (−3.0, 0.5)
**Olive Oil (g/day)**	30.2 (15.5)	29.4 (16.9)	−6.1 (−13.2, 1.1)	−4.7 (−8.5, −0.9) *	1.4 (−6.4, 9.1)
**Sweets (g/day)**	120.9 (75.6)	100.4 (70.5)	−57.6 (−91.1, −24.2) **	−48.5 (−63.6, −33.4) ***	9.1 (−23.1, 41.3)
**MVPA (min/day)**	45.7 (23.8)	43.7 (23.9)	0.6 (−10.5, 11.6)	5.5 (0.6, 10.3) *	4.9 (−5.6, 15.3)

Abbreviations: BMI, body mass index; BMI-SDS, standard deviation score for BMI; BP, blood pressure; MVPA, moderate-to-vigorous physical activity; PA, physical activity; SD, standard deviation; CI, confidence interval. * *p*-value < 0.05, ** *p*-value < 0.01 and *** *p*-value < 0.001.

**Table 2:** Change in daily macro- and micronutrient intake after lifestyle intervention in children with abdominal obesity.

	Baseline				
			Changes within group after eight weeks		
	Usual Care Group (*n* = 26)	Intensive Care Group (*n* = 81)	Usual Care Group (*n* = 26)	Intensive Care Group (*n* = 81)	Changes between Groups (Intensive vs. Usual Care)
	mean (SD)		mean (95% CI)		mean (95% CI)
**Carbohydrate (g/day)**	317.1 (83.2)	298.2 (80.1)	−91.3 (−124.4, −58.3) ***	−71.7 (−88.96, −54.50) ***	19.6 (−15.7, 54.9)
**Fibre (g/day)**	21.8 (6.0)	23.6 (7.5)	2.1 (−1.9, 6.0)	3.7 (1.8, 5.6) ***	1.6 (−2.4, 5.6)
**Protein (g/day)**	105.7 (18.0)	110.8 (20.2)	−14.6 (−23.7, −5.6) **	−11.1 (−16.1, −6.1) ***	3.6 (−6.5, 13.7)
**Total Fat (g/day)**	115.4 (29.9)	116.1 (33.0)	−38.0 (−49.7, −26.4) ***	−44.4 (−51.9, −36.9) ***	−6.4 (−21.1, 8.4)
**SFA (g/day)**	34.9 (10.7)	35.3 (13.5)	−12.0 (−16.3, −7.8) ***	−15.3 (−18.7, −12.0) ***	−3.3 (−9.5, 2.9)
**MUFA (g/day)**	50.5 (13.9)	51.6 (16.6)	−14.0 (−20.8, −7.2) ***	−19.2 (−23.6, −14.8) ***	−5.2 (−13.7, 3.2)
**PUFA (g/day)**	15.5 (6.1)	14.9 (5.4)	−4.6 (−7.3, −1.8) **	−6.1 (−7.4, −4.7) ***	−1.5 (−4.3, 1.3)
**Trans Fat (g/day)**	0.8 (0.5)	1.0 (0.5)	−0.4 (−0.6, −0.2) ***	−0.6 (−0.7, −0.4) ***	−0.2 (−0.4, 0.1)
**Cholesterol (mg/day)**	435.5 (106.1)	444.5 (115.9)	−72.7 (−110.5, −34.9) ***	−78.0 (−103.5, −52.6) ***	−5.4 (−54.7, 43.9)
**Calcium (mg/day)**	1208.2 (315.3)	1203.4 (361.7)	−165.7 (−324.1, −7.4) *	35.5 (−53.8, 124.9)	201.3 (22.0, 380.6) *
**Iron (mg/day)**	17.8 (3.9)	18.4 (4.0)	−2.3 (−4.3, −0.4) *	−2.6 (−3.5, −1.7) ***	−0.3 (−2.2, 1.7)
**Iodine (μg/day)**	341.8 (126.2)	315.5 (150.4)	−4.3 (−66.2, 57.6)	104.1 (67.3, 141.0) ***	108.4 (35.3, 181.5) **
**Magnesium (mg/day)**	383.6 (83.7)	400.5 (92.3)	−23.6 (−67.4, 20.2)	−2.6 (−26.1, 20.9)	21.0 (−26.7, 68.7)
**Zinc (mg/day)**	13.5 (2.5)	14.5 (3.3)	−1.8 (−3.3, −0.4) *	−2.1 (−2.9, −1.2) ***	−0.2 (−1.9, 1.5)
**Sodium (mg/day)**	2914.1 (655.7)	3098.7 (899.8)	−750.8 (−1023.8, −477.7) ***	−936.4 (−1155.9, −717.0) ***	−185.6 (−599.9, 228.7)
**Potassium (g/day)**	4.2 (1.0)	4.4 (1.0)	−0.3 (−0.8, 0.2)	0.1 (−0.1, 0.4)	0.4 (−1.0, 0.9)
**Phosphorus (mg/day)**	1799.9 (339.5)	1868.2 (405.3)	−191.4 (−337.9, −44.9) *	−9.3 (−106.7, 88.1)	182.1 (−7.0, 371.3)
**Selenium (μg/day)**	101.1 (30.2)	106.7 (34.4)	−10.3 (−23.9, 3.3)	−5.7 (−14.3, 2.9)	4.6 (−12.2, 21.4)
**Thiamine (mg/day)**	2.4 (0.7)	2.4 (0.6)	0.1 (−0.4, 0.5)	0.1 (−0.1, 0.2)	−0.0 (−0.4, 0.4)
**Riboflavin (mg/day)**	2.2 (0.5)	2.3 (0.6)	−0.2 (−0.4, 0.1)	0.1 (−0.1, 0.2)	0.3 (−0.0, 0.06)
**Niacin (mg/day)**	41.7 (8.3)	45.0 (8.9)	−2.1 (−6.5, 2.3)	−2.4 (−4.5, −0.2) *	−0.3 (−4.8, 4.3)
**Vitamin B6 (mg/day)**	2.3 (0.5)	2.4 (0.6)	−0.0 (−0.3, 0.3)	0.1 (−0.0, 0.2)	0.1 (−0.2, 0.4)
**Folate (μg/day)**	344.1 (101.6)	364.5 (99.1)	−2.3 (−52.9, 48.3)	21.2 (−2.8, 45.1)	23.5 (−26.8, 73.8)
**Vitamin B12 (μg/day)**	8.4 (4.4)	8.9 (3.7)	0.1 (−1.4, 1.5)	1.4 (0.5, 2.2) **	1.3 (−0.4, 3.0)
**Vitamin C (mg/day)**	187.0 (73.2)	189.2 (71.3)	−0.5 (−39.2, 38.2)	23.6 (4.2, 43.0) *	24.1 (−15.9, 64.1)
**Vitamin A (μg/day)**	1411.8 (1199.8)	1287.0 (768.5)	−276.9 (−735.3, 181.5)	6.8 (−15.0, 163.3)	283.7 (−87.1, 654.5)
**Vitamin D (μg/day)**	5.1 (3.2)	5.1 (2.8)	−0.0 (−1.3, 1.3)	1.7 (0.9, 2.5) ***	1.7 (0.1, 3.4) *
**Vitamin E (mg/day)**	10.5 (4.1)	10.8 (4.5)	−3.1 (−4.4, −1.7) ***	−2.8 (−3.7, −2.0) ***	0.2 (−1.4, 1.9)

Abbreviations: SFA, saturated fatty acid; MUFA, monounsaturated fatty acid; PUFA, polyunsaturated fatty acid; SD, standard deviation; * *p*-value < 0.05, ** *p*-value < 0.01 and *** *p*-value < 0.001.

**Table 3:** Percentage of energy from macronutrients, and proportion (%) of individuals within the acceptable macronutrient distribution range (AMDR).

		Baseline				
	AMDR			Changes within group after eight weeks		
		Usual Care Group (*n* = 26)	Intensive Care Group (*n* = 81)	Usual Care Group (*n* = 26)	Intensive Care Group (*n* = 81)	Changes between Groups (Intensive vs. Usual Care)
		mean (SD) or percentage		mean (95% CI)		mean (95% CI)
Energy (kcal/day)		2729.4 (572.6)	2681.7 (585.6)	−766.1 (−997.9, −534.3) ***	−731.1 (862.4, −599.7) ***	35.0 (−228.3, 298.4)
Carbohydrates (% Energy)		46.3 (5.3)	44.3 (6.3)	−0.1 (−2.4, 2.2)	2.0 (0.6, 3.4) **	2.1 (−0.7, 4.9)
**% Within AMDR**	45–65	54	47	0	11	11
Protein (% Energy)		15.7 (1.9)	16.8 (2.4) *	3.1 (2.1, 4.0) ***	3.8 (3.2, 4.4) ***	0.8 (−0.4, 1.9)
**% Within AMDR**	10–30	100	100	0	0	0
Fat (% Energy)		38.0 (5.3)	38.9 (6.1)	−3.0 (−5.4, −0.6) *	−5.8 (−7.3, −4.3) ***	−2.9 (−5.8, 0.1)
**% Within AMDR**	25–35	31	30	27 *	38 ***	11

Abbreviations: AMDR, acceptable macronutrient distribution range; SD, standard deviation; CI, confidence interval. * *p*-value < 0.05, ** *p*-value < 0.01 and *** *p*-value < 0.001.

**Table 4:** Effect of lifestyle intervention on the proportion of individuals with inadequate intakes under the EAR/AI **^#^** or above UL by usual and intensive care group.

	Usual Care Group (*n* = 26)				Intensive Care Group (*n* = 81)			
	<% EAR		>% UL		<% EAR		>% UL	
	Baseline	8 Weeks	Baseline	8 Weeks	Baseline	8 Weeks	Baseline	8 Weeks
**Calcium (mg/day)**	23.1	42.3	-	-	37.0	29.6	-	-
**Iron (mg/day)**	-	-	-	-	-	-	-	-
**Iodine (μg/day)**	-	-	7.7	-	2.5	-	4.9	7.4
**Magnesium (mg/day)**	7.7	11.5	73.1	65.6	6.2	2.5	70.4	76.5
**Zinc (mg/day)**	-	-	19.2	7.7	-	-	8.6	3.7
**Sodium (mg/day) ^#^**	-	7.7	92.3	53.9 **	2.5	9.9	86.4	39.5 ***
**Potassium (g/day) ^#^**	53.9	80.8			59.3	58.0		
**Phosphorus (mg/day)**	-	-	-	-	-	-	-	-
**Selenium (μg/day)**	-	-	-	-	1.2	1.2	-	-
**Thiamine (mg/day)**	-	-			-	-		
**Riboflavin (mg/day)**	-	-			-	-		
**Niacin (mg/day)**	-	-	96.2	96.2	-	-	100.0	98.8
**Vitamin B6 (mg/day)**	-	-	-	-	-	-	-	-
**Folate (μg/day)**	23.1	19.2	11.5	11.5	18.5	11.1	9.9	6.2
**Vitamin B12 (μg/day)**	-	-			-	-		
**Vitamin C (mg/day)**	-	-	-	-	-	-	-	-
**Vitamin A (μg/day)**	3.9	15.4	38.5	15.4	7.4	1.2 §§	22.2	18.5
**Vitamin D (μg/day)**	88.5	92.3	-	-	92.5	71.6 **§	-	-
**Vitamin E (mg/day)**	30.8	65.4 **	-	-	45.7	77.8 ***	-	-

Abbreviations: EAR, estimated average requirement; **^#^** AI, adequate intake; UL, upper level of intake. * § values obtained from McNemar test. *, significant difference between baseline and eight-week inadequate intake in obese children distributed by the intervention. **§**, significant difference for the comparison of change on inadequate intake between usual and intensive care group. There were no significant differences between characteristics at baseline.

**Table 5:** Change in diet quality indices after lifestyle intervention in children with abdominal obesity.

	Baseline				
			Changes within group after eight weeks		
	Usual Care Group (*n* = 26)	Intensive Care Group (*n* = 81)	Usual Care Group (*n* = 26)	Intensive Care Group (*n* = 81)	Changes between Groups (Intensive vs. Usual Care)
	mean (SD)		mean (95% CI)		mean (95% CI)
**Total Diet Quality Index** (DQI, −33% to 100%)	26.3 (9.0)	33.1 (7.4)	6.8 (2.2, 11.3) **	12.1 (10.0, 14.2) ***	5.3 (0.9, 9.8) *
**Dietary Quality DQI** (DQ, −100% to 100%)	49.5 (18.5)	71.9 (14.1)	22.3 (15.5, 29.2) ***	29.5 (25.8, 33.2) ***	7.2 (−0.3, 14.7)
**Dietary Diversity DQI** (DD, 0 to 100%)	25.5 (13.9)	19.2 (14.7)	−6.3 (−15.0, 2.5)	1.4 (−2.8, 5.5)	7.6 (−1.1, 16.3)
**Dietary Equilibrium DQI** (DA, 0 to 100%)	3.9 (5.0)	8.1 (4.0)	4.2 (2.2, 6.3) ***	5.4 (4.2, 6.6) ***	1.2 (−1.2, 3.6)
**Healthy Lifestyle Diet Index ^#^** (HLDI, 0 to 38 points)	18.3 (2.7)	19.7 (2.8)	1.4 (−0.1, 2.9)	4.1 (3.3, 4.9) ***	2.7 (1.0, 4.3) **
**Mediterranean Diet Quality Index** (KIDMED, 0–12 points)	5.2 (1.8)	7.2 (1.6)	2.0 (0.9, 3.0) ***	3.0 (2.5, 3.5) ***	1.0 (−0.1, 2.1)

Abbreviations: SD, standard deviation; CI, confidence interval. * *p*-value < 0.05, ** *p*-value <0.01 and *** *p*-value <0.001. ^#^ Index modified without daily TV viewing and computer game playing time.
